# Comparative study of esketamine and racemic ketamine in treatment-resistant depression: Protocol for a non-inferiority clinical trial

**DOI:** 10.1097/MD.0000000000012414

**Published:** 2018-09-21

**Authors:** Fernanda S. Correia-Melo, Gustavo C. Leal, Michelle S. Carvalho, Ana Paula Jesus-Nunes, Carolina B.N. Ferreira, Flávia Vieira, Guilherme Magnavita, Lucas A.S. Vale, Rodrigo P. Mello, Carolina Nakahira, Felipe C. Argolo, Tanise Cardoso, Cezar D.S. Souza, Ana Teresa C. Fontes, Marcelo B. Ferreira, Lucas Araújo-de-Freitas, Marco A. Tuena, Mariana V.F. Echegaray, Diogo E. Cavalcanti, Ana C. Lucchese, Igor D. Bandeira, Manuela Telles, Cássio S. Lima, Aline S. Sampaio, Samantha S. Silva, Roberta F. Marback, José A. Del-Porto, José Neander Abreu, Luciana M. Sarin, Camilla S. Paixão, Lucas P. Carvalho, Paulo R.L. Machado, Gustavo Turecki, Acioly L.T. Lacerda, Lucas C. Quarantini

**Affiliations:** a Postgraduate Program in Medicine and Health; b Psychiatry Service, University Hospital, Universidade Federal da Bahia, Salvador; c LiNC—Laboratório Interdisciplinar de Neurociências Clínicas; d Depatment of Anesthesiology; e PRODAF—Programa de Transtornos Afetivos, Universidade Federal de São Paulo, São Paulo; f Postgraduate Program in Psychology, Institute of Psychology; g Immunology Service, Universidade Federal da Bahial; h Clinical Research Laboratory (LAPEC), Gonçalo Moniz Institute, Fiocruz-Bahia, Salvador, Brazil; i McGill Group for Suicide Studies, Douglas Mental Health University Institute & Department of Psychiatry, McGill University, Montreal, Canada; j Center for Research and Clinical Trials Sinapse-Bairral, Instituto Bairral de Psiquiatria, Itapira, Brazil.

**Keywords:** clinical trial, depression, esketamine, isomerism, ketamine, treatment resistant

## Abstract

**Introduction:**

The use of ketamine as an option in the treatment of depressive disorder is growing rapidly, supported by numerous clinical trials attesting its efficacy and safety. Esketamine, the S (+) enantiomer of ketamine, is the most widely used form in the anesthetic environment in some countries, and new studies have shown that it may also be effective in depression and with better tolerability. However, no study so far has directly compared esketamine with racemic ketamine. Here we propose a protocol of a clinical trial to evaluate esketamine as a noninferior medication when compared to ketamine in the treatment of patients with treatment-resistant depression.

**Methods/design:**

This study protocol is for a randomized, controlled, double-blind noninferiority clinical trial. Subjects will be 18 years or older, with major depression characterized as treatment-resistant. Participants will receive a single infusion of either esketamine (0.25 mg/kg) or ketamine (0.5 mg/kg) over 40 minutes. The primary outcome will be the difference in remission rates between the 2 treatment arms at 24 and 72 hours after drug infusion. Secondary outcomes will include other timepoints, measurements of cognition, dissociation, and blood biomarkers.

**Discussion:**

A head-to-head study is the best way to evaluate whether the esketamine is in fact comparable to the racemic ketamine in terms of both efficacy and safety, and, if positive, it would be an initial step to increase the access to that type of treatment worldwide.

**Ethics and dissemination:**

The study was approved by the local Institutional Review Board (University Hospital Professor Edgard Santos—Federal University of Bahia—Number: 46657415.0.0000.0049). Subjects will only participate after voluntarily agreeing and signing the Informed Consent Form. The study findings will be published in peer-reviewed journals and presented at national and international conferences.

**Trial registration:**

This trial has been registered in the Japan Primary Registries Network (JPRN): UMIN000032355, which is affiliated with the World Health Organization.

## 1 Introduction

Major depressive disorder (MDD) is a highly prevalent clinical condition, affecting around 300 million people worldwide.^[1]^ Despite the availability of many different pharmacological treatments, about 30% of patients do not respond to available antidepressants, even after treatment with adequate dose and duration. This issue reinforces the urgent need for new therapies to manage treatment-resistant depression (TRD).^[2]^

Several clinical studies have shown that ketamine, an anesthetic drug with dissociative properties, provides a surprisingly rapid and robust antidepressant effect, when used in subanesthetic doses.^[3–6]^

The great majority of studies worldwide that evaluated the antidepressant action of ketamine have used its racemic mixture. In Brazil and some other countries, however, the most frequently available form for use in anesthesia of ketamine is its S(+) stereoisomer, also called esketamine. Previous studies have shown that the analgesic potency of S(+)-ketamine, is approximately 2 times higher than that of racemic ketamine, subjects also presented lower impairment in concentration and memory with esketamine than with the racemate.^[7]^ A study conducted by Singh et al in 2016^[8]^ evaluated the safety and efficacy of esketamine for TRD using 2 different doses (0.2 and 0.4 mg/kg), and found that both were safe and effective, but the smaller dose was better tolerated, and the most commonly reported side effects were transient dissociation, dizziness, dry mouth, and headache. Despite the possibility that esketamine is better tolerated than racemic ketamine, while maintaining the antidepressive efficacy, we found no controlled study that has conducted a drug-to-drug comparison between racemic ketamine and esketamine in humans for the treatment of depressive illness, we found only a report of 2 subjects who received both forms in different time points, favoring esketamine in terms of side effects.^[9]^

The search for biomarkers associated with ketamine treatment is another area of great interest, and many candidates are currently being studied. Reductions in brain-derived neurotrophic factor (BDNF), a protein of the neurotrophic family of growth factors,^[10]^ has been reported in different neuropsychiatric diseases.^[11–14]^ The BDNF blood levels are decreased in MDD subjects and increase after antidepressant treatment.^[15,16]^ Epigenetic mechanisms are also being investigated in individuals with MDD, especially the regulation of microRNAs level. Some studies have shown modified expression of microRNAs after antidepressant treatment.^[17,18]^ Other studies have shown that inflammation in MDD is a factor that should be evaluated and understood and it is related to the increase of proinflammatory cytokines such as interleukin 1-beta (IL-1B), interleukin 6 (IL-6), tumor necrosis factor-alpha (TNF-A), and interferon-gamma (IFN-G), among others.^[19–21]^

Expanding the knowledge about biomarkers may help to identify mechanisms involved in the antidepressant effects of ketamine and enhance the response to treatment. Also, as all the biomarkers cited above (microRNA, BDNF, cytokines and metabolomic patterns) can be measured in peripheral blood samples, the potential harm to patients is minimal, and if shown to have a clinical utility, it would be a quite valuable information.

The administration of esketamine as a form of treatment for MDD is already being practiced throughout Brazil and other countries and it apparently offers promising and safe results. By comparing these 2 forms of ketamine, we may verify the efficacy and safety of esketamine as a therapeutic option to TRD.

## 2 Methods and design

### 2.1 Study design

The present trial is a prospective, randomized, controlled, intervention, double-blind, noninferiority study, with 2 parallel groups, that will include a total of 96 individuals, with a follow-up duration of 7 days after randomization. Patients will maintain naturalistic treatments (not changing dose or type of medicine) until 15 days before the randomization; this should minimize potential biases caused by differences between groups, but they may not have new drugs included in their treatment regimen during the study week (except for non-benzodiazepine sleep inducers). After completion of the study week, patients will continue under the most convenient treatment regimen.

### 2.2 Study settings and participants

The study will be performed in the Psychiatric Ward of Professor Edgard Santos University Hospital (HUPES—Federal University of Bahia, in the city of Salvador) and São Paulo Hospital (Federal University of São Paulo, in the city of São Paulo) in Brazil. Both cities are among the largest urban areas in the country, with an ample variety of social, cultural, and ethnic characteristics, adequately reflecting the country's diverse population. Participants will also be referred from low and high complexity outpatient services, both public and private.

The study was approved by the local Institutional Review Board (Professor Edgard Santos University Hospital—Federal University of Bahia—Number: 46657415.0.0000.0049). Current protocol version: 1.4, with past and possible future modifications always reported to the local IRB. Subjects will only participate after voluntarily agreeing and signing the informed consent form. The institutions in which the study procedures will be conducted will provide necessary hospital structure, drugs, and human resources. Additionally, a grant provided by the Programa de Pesquisa para o SUS (PPSUS) through Fundação de Amparo à Pesquisa na Bahia (FAPESB), a local public research-funding organization (Grant identification number: 5301/2017), will guarantee further resources. The funders had no role in study design, data collection, and analysis, decision to publish, or preparation of the manuscript.

This trial has been registered in the Japan Primary Registries Network (JPRN): UMIN000032355, which is affiliated with the World Health Organization (WHO).

Subjects will be males and females, aged 18 years or older, meeting all following inclusion criteria at the time of randomization: DSM-IV (Diagnostic and Statistical Manual of Mental Disorders version IV) MDD diagnosis confirmed with the Brazilian version of the Mini International Neuropsychiatric Interview 5.0.0 (MINI-Plus),^[22]^ characterized as TRD, defined by therapeutic failure after at least 1 adequate antidepressant treatment for at least 12 weeks. The exclusion criteria will be: concomitant treatment with electroconvulsive therapy, diagnosis of a psychotic disorder, mental retardation or dementia, unstable heart disease, and current illicit drug use.

All study-related information will be stored securely at the study sites in areas with limited access. Laboratory specimens will be identified by a coded identification to maintain participant confidentiality.

### 2.3 Baseline assessment

At baseline, all participants will be assessed with a sociodemographic questionnaire and eligibility criteria will be analyzed using MINI-Plus 5.0.0, intelligence evaluation, and treatment-resistance criteria evaluation.

### 2.4 Primary outcome measure

The primary outcome will be the difference in remission rates between the 2 treatment arms at 24 and 72 hours. Remission is defined as a Montgomery-Åsberg Depression Rating Scale (MADRS)^[23]^ score of ≤7. Baseline MADRS will be performed at the most within 7 days before the intervention, together with other baseline assessments, and repeated 24 and 72 hours after the intervention to evaluate remission. As the primary outcome is measured shortly after the intervention, we expect a low level of data loss.

### 2.5 Intervention and group assignment

Participants will be submitted to a single dose of one of the 2 drugs in the study:

- Ketamine racemic mixture (or ketamine hydrochloride):
  ∘ Dose: 0.5 mg/kg of body weight
  ∘ Commercial brand: Clortamina from Biochimico
  ∘ Dosing form: injectable solution, 10 mL ampoules, 50 mg/mL
- Esketamine (or S[+]-ketamine, or dextroketamine hydrochloride)
  ∘ Dose: 0.25 mg/kg of body weight
  ∘ Commercial brand: Ketamin from Cristalia
  ∘ Dosing form: injectable solution, 2 mL ampoules, 50 mg/mL

Both drugs will be diluted in 100 mL of saline solution and administered intravenously with an infusion pump for a total duration of 40 minutes. The dose of 0.5 mg/kg of ketamine is the most commonly used in trials for depression,^[3]^ as for esketamine, we chose the 0.25 mg/kg dose, as it is considered 2 times more potent than racemic ketamine as an anesthetic.^[7]^ Previous experience of our group pointed that a faster infusion of esketamine, 10 minutes, is associated with reduced tolerability because of intense dissociation,^[24,25]^ so we chose to use the standard 40 minutes’ duration.^[3]^

Participants will be randomized into esketamine and ketamine groups, on a 1:1 ratio, through an electronic randomization software http://www.randomizer.org platform. The only professionals with knowledge of the drug being infused are the investigator responsible for the allocation process, who will be only one for both centers, and a nurse responsible for drug preparation, one for each center. They do not participate in any clinical evaluations. As both drugs will be diluted in a saline bottle, it will not be possible to identify them visually. It is possible that the two forms of ketamine differ in terms of adverse effects, especially psychotomimetic, but those effects are very idiosyncratic and will help maintain the blinding of the study.

### 2.6 Secondary outcome measures

MADRS will also be performed 7 days after the intervention to access duration of remission. Treatment response, defined as ≥ 50% reduction in baseline score, will also be accessed with MADRS. The Global Clinical Impression (CGI) scale,^[26]^ will be an additional measure of efficacy.

Previous dissociative experiences will be assessed with a questionnaire, the Dissociative Experience Scale (DES),^[27]^ performed before the administration of the drug. To evaluate dissociative/confusional symptoms intensity, the Clinician-Administered Dissociative States Scale (CADSS)^[28,29]^ will be used. Assessment with CADSS will be initiated 20 minutes after the beginning of the infusion, as it is when dissociative symptoms start to rise.

Columbia-Suicide Severity Rating Scale (C-SSRS)^[30]^ will be used for assessing suicidality, and will be performed at baseline, then 24 hours, 72 hours, and 7 days after the infusion. Young Mania Rating Scale^[31]^ will only be performed in case of evident manic symptoms at any of the evaluation days. Finally, Medication Adherence Rating Scale (MARS)^[32]^ will be used to measure the psychiatric drugs adherence and performed at enrollment day.

To examine the relationship between clinical response and molecular and epigenetic mechanisms approximately 20 mL of blood will be collected to analyze serum levels of microRNA-206 and microRNA-1202, BDNF, IL-1B, IL-6, TNF-A, and other elements for the metabolomics profile at different timepoints.

Neuropsychological evaluations will be performed to measure cognitive functions: intelligence, executive functions (inhibitory control, cognitive flexibility, verbal fluency), attention, processing speed, memory and working memory (verbal and visual). Aspects related to personality will be analyzed based on the theory of Goldberg's Big Five factors of personality, using the NEO-FFI-R (NEO Five Factor Inventory Revised), a scale composed of 60 items that evaluate the five dimensions of the personality.^[33]^

The schedule of assessments and other procedures is summarized in Table 1.

**Table 1:**
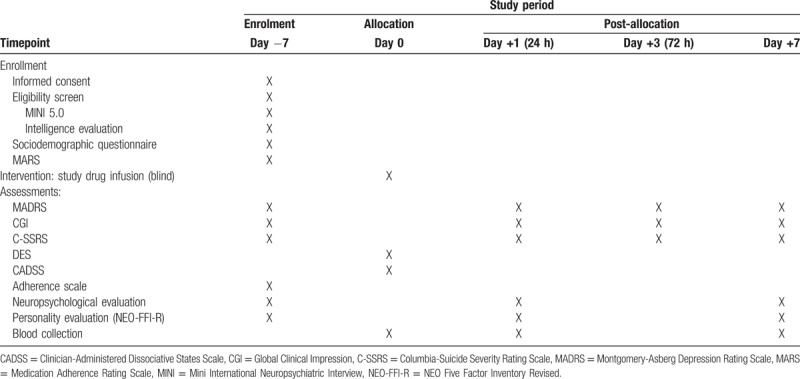
Participant timetable.

### 2.7 Safety and monitoring

During the infusion, the patient will be monitored every 5 minutes for blood pressure (BP) levels, heart rate, peripheral oxygen saturation, respiratory pattern and level of consciousness. The patient will also be asked to report any other possible adverse event, not only during the intervention procedure, but at the subsequent visits at 24 hours, 72 hours, and 7 days after infusion. In case of elevated BP <30% of the pre-infusion levels, the infusion will be paused for 5 minutes and the patient evaluated for acute target organ damage. If there is no sign of acute complication and the BP levels drop, the infusion may continue and be completed.

Serious adverse events will be reported to the Ethics Committee of the hospital. The association of an event to the study drug will be evaluated based on a temporal and biological correlation analyses. As the intervention is conducted only once, blind will only be broken if strictly necessary and after discussion with the study senior coordinator.

## 3 Statistical considerations

We selected a noninferiority margin of 20% based on clinical judgment. Assuming the control group would have a remission proportion of 30% based on literature data. Our board of experts deemed this a reasonable threshold to clinically consider using an intervention in TRD.

Assuming the intervention group to have a similar remission proportion of 30%, we calculated a sample size of 130 people (in both groups) would be needed to achieve 80% power with an alpha of 5% in the one-sided hypothesis test for efficacy. Owing to logistic constraints, we are limited to 96 patients, which brings our power down to 66%, keeping all other parameters constant. We decided to run the study even though underpowered upon starting the protocol, and to reassess power during the interim analysis.

All analyses will be conducted within the intention-to-treat (ITT) perspective. Intention-to-treat analysis can bias results in studies with active controls, but as the study intervention is a 1-time in-hospital drug infusion we expect no deviations from the randomized treatment. Summary statistics will be presented as means and standard deviations, medians, and interquartile ranges or frequencies based on variable distribution.

All the main analyses will be performed with univariate hypothesis tests. The main outcome (remission on the MADRS scale at 24 hours after infusion) will be analyzed by calculating a point estimate for the difference in remission proportion among the intervention and active control groups, and then by building a 1-sided 95% confidence interval. The null hypothesis will be rejected if the margin of this confidence interval is equal to or smaller than the noninferiority margin. All other secondary efficacy outcomes will also be analyzed by computing a bound for a 1-sided 95% confidence interval for parameter difference (means or proportion depending on variable distribution), with sensitivity analysis with 2-sided traditional hypothesis testing. All safety secondary outcomes will be compared with 2-sided hypothesis testing based on variable distribution (T test for normally distributed variables, *χ*^2^ or Fisher exact test for proportions).

We will perform an interim analysis for efficacy, futility, and safety upon reaching 50% of target enrollment (48 patients). We will adjust our alpha according to the Maybittle-Peto rule in the interim analysis for efficacy to avoid “alpha spending.” Futility will be assessed by calculating conditional power based on an exact method for calculating the joint probability of all scenarios of the final 2 × 2 table that reject the null hypothesis using binomial distributions and an indicator function for null hypothesis rejection.

## 4 Discussion

The effect of the discovery of ketamine as a treatment for depressive disorder, including treatment resistant cases, is immense, but many questions still remain about its clinical applicability and its mechanism of action. One obstacle for the more widespread use of ketamine is that most clinical studies until now have used the racemic form, whereas some countries only have the S (+) isomer available. A head-to-head study is the best way to evaluate whether the esketamine is in fact comparable to the racemic ketamine in terms of both efficacy and safety, and, if positive, it would be an initial step to increase the access to that type of treatment worldwide. Also, it is possible that esketamine is associated with less dissociative symptoms than ketamine,^[9,34]^ what would be a great advantage, as dissociation might be the most significant adverse effect of ketamine treatment, and only a direct comparison between both forms can provide a sound answer to this question.^[35]^ Previous reports corroborate the hypothesis that esketamine produces less dissociative action, as schizophrenia subjects and depressive psychotic individuals not only tolerated well but also showed relevant clinical improvement^[36,37]^

Another important aspect of a head-to-head comparison between the 2 forms of ketamine is that it might shed more light in the quest for understanding the biological mechanisms by which ketamine exerts its antidepressant effect. Our study includes a series of biomarker measurements; different patterns of these markers between esketamine and ketamine can suggest possible biological pathways of action.

### 4.1 Trial status

This trial is currently recruiting participants. Recruitment began on March, 2017. We expect the recruitment phase to be completed by the end of 2018. The study findings will be published in peer-reviewed journals and presented at national and international conferences when completed.

## Acknowledgments

The authors thank Dr. Collen K Loo for the interest and help in reviewing this protocol, and also thank Mikar Lopez who performed the review as a native English speaker.

## Author contributions

**Conceptualization:** Fernanda S Correia-Melo, Gustavo Carneiro-Gomes Leal, Lucas C Quarantini.

**Data curation:** Fernanda S Correia-Melo, Gustavo Carneiro-Gomes Leal, Flávia Vieira, Felipe C. Argolo, Tanise Cardoso, Ana-Teresa Caliman Fontes, Lucas Araújo-de-Freitas, Mariana Echegaray, Diogo E Cavalcanti, Cássio S Lima, Roberta Marback, José Neander Abreu, Acioly LT Lacerda.

**Formal analysis:** Fernanda S Correia-Melo, Gustavo Carneiro-Gomes Leal, Flávia Vieira, Guilherme Magnavita, Felipe C. Argolo, Lucas C Quarantini.

**Funding acquisition:** Lucas C Quarantini.

**Investigation:** Fernanda S Correia-Melo, Gustavo Carneiro-Gomes Leal, Michelle S Carvalho, Ana Paula Jesus-Nunes, Carolina B N Ferreira, Flávia Vieira, Lucas A S Vale, Rodrigo P Mello, Carolina Nakahira, Tanise Cardoso, Cezar D S Souza, Ana-Teresa Caliman Fontes, Marcelo B. Ferreira, Marco A Tuena, Mariana Echegaray, Ana C Lucchese, Igor D Bandeira, Aline Santos Sampaio, Samantha Siqueira Silva, José A Del-Porto, Luciana M Sarin, Camilla Sampaio Paixão, Lucas Pedreira de Carvalho, Acioly LT Lacerda.

**Methodology:** Fernanda S Correia-Melo, Gustavo Carneiro-Gomes Leal, Ana Paula Jesus-Nunes, Guilherme Magnavita, Rodrigo P Mello, Tanise Cardoso, Ana-Teresa Caliman Fontes, Cássio S Lima, José Neander Abreu, Lucas Pedreira de Carvalho, Paulo RL Machado, Acioly LT Lacerda, Lucas C Quarantini.

**Project administration:** Fernanda S Correia-Melo, Gustavo Carneiro-Gomes Leal, Lucas C Quarantini.

**Resources:** Ana-Teresa Caliman Fontes, Lucas Araújo-de-Freitas, Mariana Echegaray, Cássio S Lima, Roberta Marback, José Neander Abreu, Lucas C Quarantini.

**Supervision:** Fernanda S Correia-Melo, José Neander Abreu, Acioly LT Lacerda, Lucas C Quarantini.

**Validation:** Lucas C Quarantini.

**Visualization:** Gustavo Carneiro-Gomes Leal, Guilherme Magnavita, Tanise Cardoso, Ana-Teresa Caliman Fontes, Lucas Araújo-de-Freitas, Lucas C Quarantini.

**Writing - original draft:** Fernanda S Correia-Melo, Gustavo Carneiro-Gomes Leal, Ana Paula Jesus-Nunes, Guilherme Magnavita, Rodrigo P Mello, Acioly LT Lacerda, Lucas C Quarantini.

**Writing - review & editing:** Fernanda S Correia-Melo, Gustavo Carneiro-Gomes Leal, Ana Paula Jesus-Nunes, Guilherme Magnavita, Rodrigo P Mello, Felipe C. Argolo, Roberta Marback, Gustavo Turecki, Acioly LT Lacerda, Lucas C Quarantini.
